# Effects of linagliptin on endothelial function and postprandial lipids in coronary artery disease patients with early diabetes: a randomized, placebo-controlled, double-blind trial

**DOI:** 10.1186/s12933-018-0716-x

**Published:** 2018-05-17

**Authors:** Norbert J. Tripolt, Felix Aberer, Regina Riedl, Jasmin Url, Gudrun Dimsity, Andreas Meinitzer, Tatjana Stojakovic, Faisal Aziz, Ronald Hödl, Gabriele Brachtl, Dirk Strunk, Marianne Brodmann, Franz Hafner, Harald Sourij

**Affiliations:** 1 Cardiovascular Diabetology Research Group, Division of Endocrinology and Diabetology, Department of Internal Medicine, Medical University of Graz, Auenbruggerplatz 15, 8036 Graz, Austria; 2 Division of Angiology, Department of Internal Medicine, Medical University of Graz, Graz, Austria; 3 Institute for Medical Informatics, Statistics and Documentation, Medical University of Graz, Graz, Austria; 4 Clinical Institute of Medical and Chemical Laboratory Diagnostics, Medical University of Graz, Graz, Austria; 5 Center for Cardiovascular Rehabilitation St. Radegund, St. Radegund, Austria; 6 Experimental & Clinical Cell Therapy Institute, Spinal Cord & Tissue Regeneration Center Salzburg, Paracelsus Private Medical University, Salzburg, Austria; 7 Center for Biomarker Research in Medicine, CBmed, Graz, Austria

**Keywords:** Linagliptin, Flow mediated dilatation, RCT, DPP-4 inhibitor, Type 2 diabetes, Coronary artery disease, Endothelial function

## Abstract

**Background:**

Early glucose lowering intervention in subjects with type 2 diabetes mellitus was demonstrated to be beneficial in terms of micro- and macrovascular risk reduction. However, most of currently ongoing cardiovascular outcome trials are performed in subjects with manifest atherosclerosis and long-standing diabetes. Therefore, the aim of this study is to investigate the effects of the dipeptidylpeptidase-4 inhibitor linagliptin in subjects with coronary artery disease (CAD) but early type 2 diabetes mellitus (T2DM) on a set of cardiovascular surrogate measurements.

**Methods:**

In this randomized, placebo-controlled, double-blind, single-center study, we included subjects with early diabetes (postchallenge diabetes (2 h glucose > 200 mg/dl) or T2DM treated with diet only or on a stable dose of metformin monotherapy and an HbA1c < 75 mmol/mol) and established CAD. Participants were randomized to receive either linagliptin (5 mg) once daily orally or placebo for 12 weeks. The primary outcome was the change in flow mediated dilatation (FMD). The secondary objective was to investigate the effect of linagliptin treatment on arginine bioavailability ratios [Global arginine bioavailability ratio (GABR) and arginine to ornithine ratio (AOR)]. Arginine, ornithine and citrulline were measured in serum samples with a conventional usual amino acid analysis technique, involving separation of amino acids by ion exchange chromatography followed by postcolumn continuous reaction with ninhydrin. GABR was calculated by l-arginine divided by the sum of (l-ornithine plus l-citrulline). The AOR was calculated by dividing l-arginine by l-ornithine levels. Group comparisons were calculated by using a two-sample t-test with Satterthwaite adjustment for unequal variances.

**Results:**

We investigated 43 patients (21% female) with a mean age of 63.3 ± 8.2 years. FMD at baseline was 3.5 ± 3.1% in the linagliptin group vs. 4.0 ± 2.9% in the placebo group. The change in mean FMD in the linagliptin group was not significantly different compared to the change in the placebo group (0.43 ± 4.84% vs. − 0.45 ± 3.01%; p = 0.486). No significant improvements were seen in the arginine bioavailability ratios (GABR; p = 0.608 and AOR; p = 0.549).

**Conclusion:**

Linagliptin treatment in subjects with CAD and early T2DM did not improve endothelial function or the arginine bioavailability ratios.

*Trial registration* ClinicalTrials.gov, NCT02350478 (https://clinicaltrials.gov/ct2/show/NCT02350478)

**Electronic supplementary material:**

The online version of this article (10.1186/s12933-018-0716-x) contains supplementary material, which is available to authorized users.

## Background

Patients with type 2 diabetes (T2DM) are at increased risk of macrovascular events as well as microvascular complications [1]. Endothelial dysfunction represents an early step in the process of atherosclerosis and moreover serves as a surrogate for future cardiovascular events. We and others have shown previously, that endothelial dysfunction is present in patients with coronary artery disease and early diabetes and can be improved by pharmacological intervention [2].

Dipeptidylpeptidase-4 (DPP-4) inhibitors increase endogenous glucagon like-peptide-1 (GLP-1) levels leading to an insulin release from pancreatic beta-cells and suppression of glucagon secretion from alpha cells in a glucose dependent manner [3]. Hence, this drug class was demonstrated to lower both, fasting and post-challenge or postmeal glucose levels and consequently glycosylated haemoglobin (HbA1c) and is well tolerated.

Three cardiovascular outcome trials with the DPP-4 inhibitors alogliptin, saxagliptin and sitagliptin were published and demonstrated cardiovascular safety of those molecules as compared to usual diabetes care without DPP-4 inhibitors [4–6]. Two outcome trials with linagliptin (CAROLINA [7] and CARMELINA—NCT01897532) are ongoing. All published studies included subjects with longstanding diabetes (median duration between 7.1 and 11.6 years) and it has been argued that results might be different in subjects with shorter diabetes duration.

A well-known and validated cardiovascular surrogate parameter for future cardiovascular events is the dysfunction of endothelium, characterized by impairment of relaxation of the arteries in response to increased shear stress [8]. Mechanistic animal and in vitro data demonstrated, that DPP-4 inhibitions leads to improved vascular stiffening, endothelial function, vascular remodelling, suggesting potential cardiac, renal and neurologic benefits of this drug class [9, 10]. Reduction in oxidative stress, inflammatory responses or apoptosis and beneficial effects on the AGE/RAGE axis were proposed as possible molecular mechanisms leading to the vascular and renal effects observed [11].

Data on the effect of DPP-4 inhibitors on endothelial function are conflicting. While Nakamura et al. [12] demonstrated an improvement of endothelial function with Sitagliptin, Ayaori and contributors [13] showed a worsening of endothelial function using the same drug. However, Jax et al. demonstrated a neutral effect on macrovascular function [14]. However, not all these trials were performed in a placebo-controlled fashion.

Therefore, we performed a randomized, placebo-controlled, double-blind trial investigating the effect of the DPP-4 inhibitor linagliptin on endothelial function and further biochemical markers of vascular function and effects on postprandial lipids of the DPP-4 inhibitor linagliptin in early stages of T2DM.

## Methods

We conducted a prospective, single-center, double-blind, randomized placebo-controlled trial at the Medical University of Graz, Austria (ClinicalTrials.gov, NCT02350478). The study was approved by the local Ethics Committee of the Medical University of Graz (25–295 ex 12/13) and was performed in accordance with the principles of the Declaration of Helsinki, good clinical practice guidelines, and the standard operating procedures of the sponsor. Written informed consent was obtained from all participants prior to enrolment.

Detailed information regarding the design and methods of this study were described previously [15]. Briefly, patients with early T2DM and manifest coronary atherosclerosis (diagnosed either by coronary angiography or coronary computer tomography), aged between 40 and 80 years, with either a 2 h-glucose value above 200 mg/dl after 75 g glucose load or a glycated haemoglobin (HbA1c) level between 42 and 75 mmol/mol while on diet only or metformin monotherapy, were included in the trial. Participants were randomized to receive either linagliptin [linagliptin group (LG)] or matched placebo drug [placebo group (PG)] administered orally for a duration of 12 weeks. Randomization was performed by using the web-based randomization tool “Randomizer for Clinical Trials: http://www.randomizer.at/” (Accessed 29 Jan 2018) provided by the Institute of Medical Informatics, Statistics and Documentation, Medical University of Graz. Patients were randomly assigned to one of the two arms (LG or PG, ratio 1:1). Permuted block randomization with block size 10 was used.

Mobilization of hematopoietic stem/progenitor cells (HSPC) endothelial progenitor cells (EPC) and mesenchymal stromal cells (MSC) was analysed by multi-parameter flow cytometry as described previously using heparinised peripheral blood samples [16]. Briefly, at least one million mononuclear cells per sample were analysed to determine mature cell lineage marker-negative (Lin^NEG^) CD34^+^/CD45^+^/CD90^+^/CD271^−^ HSPC, CD34^+^/CD45^−^/CD90^−^/CD271^−^ EPC and CD34^−^/CD45^−^/CD90^+^/CD271^+^ MSC based on previously published procedures [17, 18]. HSPC subpopulations were measured as Lin^NEG^/CD34^+^/CD38^−^/CD45RA^+^ lymphoid multipotent progenitors (LMPP), Lin^NEG^/CD34^+^/CD38^+^/CD10^−^/CD123^−^/CD45RA^−^ megakaryocyte-erythroid progenitors (MEP), Lin^NEG^/CD34^+^/CD38^+^/CD10^+^/CD45RA^+^ common lymphoid progenitors (CLP) and Lin^NEG^/CD34^+^/CD38^−^/CD45RA^−^ multipotent progenitors (MPP) [19].

### Statistical analysis

For sample size calculation a relative improvement of the FMD of 30% in the linagliptin group and no changes from baseline to week 12 in the placebo group were assumed. For a total sample size of 42 subjects and based on an absolute difference between the two groups in the FMD change from baseline to week 12 of 1.5% with a SD of 1.5%, a power of approximately 90% could be achieved (two-sided test with alpha 5%). All statistical analyses were performed with SAS^®^ software version 9.4 (SAS Institute, Cary, NC, USA). Data are expressed as mean (± standard deviation), unless otherwise specified.

Net incremental area (net area above baseline) was calculated for c-peptide, glucose and insulin, and net decremental area (net area below baseline) for the free fatty acids based on the trapezoidal rule with baseline value as mean of the values at time points − 5 and 0 min. For the primary and secondary endpoints, group differences were analysed using analysis of covariance with the follow-up value at week 12 as dependent variable, and the baseline value and treatment group as covariates. If the assumptions of the ANCOVA were not fulfilled, parameter changes between the groups were compared by t-test or Mann–Whitney-U-test. Laboratory parameters having a skewed distribution were log-transformed. As post hoc sensitivity analysis, multiple linear regression analysis for change in FMD including age, gender, eGFR, NT-proBNP, LDL-cholesterol and systolic blood pressure at baseline was conducted. All analyses were performed on intention-to-treat (ITT) basis. Statistical significance was defined as p < 0.05.

## Results

### Study population

**Fig. 1:**
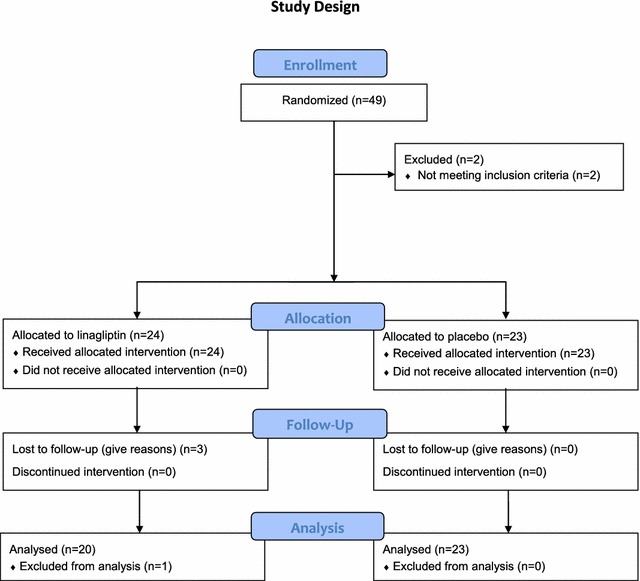
Flow diagram of the study

In total 49 randomizations were performed. After randomization, two subjects were excluded due to screening failures without receiving the study medication. Therefore 47 subjects were enrolled in the study and received the study medication. Of those, 3 subjects were lost to follow up without available follow-up data and 1 subject was excluded due to a major protocol violation prior to unblinding (Fig. 1).

In the final analysis we included 43 patients (9 females/34 males) with a mean age of 63.3 ± 8.2 years and a mean diabetes duration of 4.3 ± 5.5 years. Mean HbA1c at baseline was 52.3 ± 8.2 mmol/mol in the LG and 51.0 ± 8.3 mmol/mol in the PG, respectively. Overall, 16 (8 in the LG vs. 8 in the PG) subjects were treated with diet only, while 27 (12 in the LG vs. 15 in the PG) participants were on metformin monotherapy. No differences with respect to any of the baseline parameters were seen between PG and LG at the time of enrolment (Table 1).

**Table 1:** Baseline characteristics

	Linagliptin			Control			p-value^a^
	Baseline	3 months	Δ	Baseline	3 months	Δ	
Blood pressure systolic (mmHg)	134 ± 17	142 ± 14	8 ± 18	128 ± 18	132 ± 18	4 ± 18	0.103
Blood pressure diastolic (mmHg)	79 ± 14	81 ± 12	1 ± 14	78 ± 10	78 ± 12	0 ± 14	0.599
Low density lipoprotein (mg/dl)	66 ± 31	80 ± 34	13 ± 43	76 ± 43	78 ± 43	4 ± 23	0.408
Triglycerides (mg/dl), median (range)	145 (54 to 480)	132 (68 to 451)	− 16 (− 108 to 197)	122 (63 to 255)	146 (62 to 651)	14 (− 16 to 396)	0.173
HbA1c (mmol/mol), median (range)	50.5 (43 to 69)	49 (38 to 88)	− 2 (− 8 to 27)	51 (39 to 78)	48 (39 to 71)	0.5 (− 28 to 18)	0.029
Fasting blood glucose (mg/dl)	136 ± 41	130 ± 39	− 4 ± 18	123 ± 26	125 ± 36	4 ± 37	0.670
Aspartate-aminotransferase (U/L)	33.5 ± 14	34.9 ± 20.6	0.9 ± 21.9	27.6 ± 8.3	31.8 ± 12.9	4.7 ± 14.1	0.869
Alanine-aminotransferase (U/L)	40.4 ± 25	37.7 ± 21.7	− 3.6 ± 21.4	29 ± 12.7	29.2 ± 11.9	1.0 ± 7.4	0.905
Creatinine (mg/dL)	1.0 ± 0.3	1.0 ± 0.3	0.0 ± 0.1	1.0 ± 0.2	1.0 ± 0.2	0.0 ± 0.1	0.327
eGFR (ml/min)	76.7 ± 16.1	73.2 ± 17.6	− 2.5 ± 11.4	80.8 ± 18.0	81.3 ± 17.5	0.5 ± 8.6	0.245
NT-proBNP (pg/ml)	419 ± 528	486 ± 519	65 ± 377	228 ± 452	232 ± 589	4 ± 192	0.543
c-Reactive protein (mg/L)	3.8 ± 4.2	2.4 ± 3.0	− 1.5 ± 2.5	7.6 ± 20.4	3.3 ± 4.5	− 4.6 ± 21.2	0.548

*eGFR* estimated glomerular filtration rate, *NT-proBNP* N-terminal pro b-type natriuretic peptide

^a^p values based on analysis of covariance except for triglycerides and HbA1c (Mann–Whitney-U-test for differences). Δ reflects difference between 3 months and baseline. Data are mean ± SD unless otherwise stated

### Endothelial function

At baseline FMD measurement was 3.5 ± 3.1% in the LG and 4.0 ± 2.9% in the PG, respectively (Table 2). The increase in mean FMD in the LG (0.4 ± 4.8%) was not significantly different compared to the change in the PG (− 0.5 ± 3.0%; p = 0.486). The sensitivity analysis for change in FMD including age, gender, eGFR, NT-proBNP, LDL-cholesterol and systolic blood pressure at baseline did not change the results (data not shown). No significant improvements were observed in the change of other endothelial function parameters such as Global Arginine Bioavailability Ratio (GABR) (change − 0.11 ± 0.35 in the LG vs. − 0.06 ± 0.39 in the PG; p = 0.608), the change of the arginine-to-ornithine-ratio (AOR) (− 0.13 ± 0.45 in the LG vs. − 0.05 ± 0.53 in the PG; p = 0.549), change in asymmetric dimethylarginine (ADMA) (0.15 ± 0.22 µmol/L in the LG vs. 0.10 ± 0.14 µmol/L in the PG; p = 0.28), change of serum soluble intercellular adhesion molecule-1 ([sICAM-1]-15 (− 272 to 103) vs. − 21 (− 134 to 310) ng/ml; p = 0.903) or change of serum soluble vascular cell adhesion molecule-1 ([sVCAM-1]-34 ± 84 vs. 5 ± 130 ng/ml; p = 0.431), respectively.

**Table 2:** Effect of linagliptin treatment on primary and secondary outcome parameters

	Linagliptin			Placebo			p-value^a^
	Baseline	3 months	Δ	Baseline	3 months	Δ	
Global Arginine Bioavailability Ratio (GABR)	0.9 ± 0.36	0.81 ± 0.29	− 0.11 ± 0.35	0.93 ± 0.31	0.86 ± 0.35	− 0.06 ± 0.39	0.608
Arginine to Ornithine Ratio (AOR)	1.13 ± 0.48	1.02 ± 0.38	− 0.13 ± 0.45	1.15 ± 0.36	1.10 ± 0.47	− 0.05 ± 0.53	0.549
FMD (%)	3.5 ± 3.1	3.9 ± 3.3	0.4 ± 4.8	4.0 ± 2.9	3.5 ± 3.3	− 0.5 ± 3.0	0.486
NMD (%)	14.0 ± 5.9	13.2 ± 6.4	1.1 ± 7.4	13.6 ± 5.2	12.9 ± 4.9	− 0.7 ± 5.1	0.963
sICAM-1 (ng/ml)	929 (692 to 992)	922 (514 to 995)	− 15 (− 272 to 103)	852 (547 to 1164)	914 (597 to 1030)	− 21 (− 134 to 310)	0.903
sVCAM-1 (ng/ml)	755 ± 80	712 ± 189	− 34 ± 84	713 ± 271	716 ± 264	5 ± 130	0.431
C-peptide (mg/dl) AUC	370 ± 162	353 ± 183	− 3 ± 161	371 ± 191	342 ± 189	− 34 ± 211	0.562
Glucose (mg/dl) AUC	5739 ± 3359	4434 ± 2714	− 1135 ± 2619	4118 ± 2132	4670 ± 2636	481 ± 3185	0.456
Insulin (mg/dl) AUC	5456 ± 5074	5416 ± 3890	249 ± 4766	5765 ± 5342	6508 ± 5966	40 ± 6357	0.855
Free Fatty Acids (µmol/l) AUC	− 15.4 ± 28.7	− 15.5 ± 13.4	2.0 ± 28.4	− 19.3 ± 16.9	− 22.3 ± 19.3	− 3.1 ± 18.3	0.452

*sICAM-1* serum soluble intercellular adhesion molecule-1, *sVCAM-1* soluble vascular cell adhesion molecule-1, *AUC* area under the curve, *NMD* nitroglycerin mediated dilatation, *FMD* flow mediated dilatation

^a^p-values based on analysis of covariance except for SiCAM-1 (Mann–Whitney-U-test for differences). NET-AUC in minutes; Δ reflects difference between 3 months and baseline

### Glucose and lipid metabolism

HbA1c was significantly reduced with linagliptin treatment − 2 (− 8 to 27) mmol/mol in the LG vs. 0.5 (− 28 to 18) mmol/mol in the PG; p = 0.029). Compared with the placebo group, subjects receiving linagliptin showed a numerical, but not statistically significant reduction of the area under curve (AUC) for glucose (Table 2). AUC for insulin, C-peptide and free fatty acids were comparable between both treatment groups (Table 2).

### Ancillary study for hematopoietic stem cells

In a subset of randomly selected 8 subjects (5 in the linagliptin and 3 in the placebo group) endothelial progenitor cells (EPC) and mesenchymal stromal cells (MSC) were investigated. Neither increased nor reduced mobilization of EPCs and MSC were observed in this small subgroup (see Additional file 1: Table S1).

Serious adverse events occurred in 3 subjects in the LG vs. in 1 subject in the PG (p = 0.323), none of the events were related to the study medication.

## Discussion

We investigated the impact of the DPP-4 inhibitor linagliptin on markers of endothelial function and postprandial lipid excursions in subjects with established coronary artery disease and early diabetes treated either with diet alone or metformin monotherapy. We observed, that linagliptin neither improves flow mediated dilation or other biochemical markers of endothelial function nor postprandial lipid levels.

Animal studies suggested beneficial effects of DPP-4 inhibitors on atherosclerosis [20]. Although first human studies suggested a beneficial effect on endothelial function [12] there were others showing even a worsening of endothelial function [13], while others turned out to be neutral [14, 21].

In a recent trial, Ott et al. investigated the impact of linagliptin on renal endothelial dysfunction and their data of a 4 week trial suggest the prevention of hyperglycaemia induced impaired renal endothelial function by linagliptin [22]. In contrast to our study, they focused on renal endothelial function, while we used flow mediated vasodilation as an assessment of vascular function and the major difference is, that our cohort had established coronary atherosclerosis as an inclusion criterion. Effects of linagliptin on endothelial function in subjects without manifest atherosclerosis might be different than the neutral effects observed in our cohort.

Shigiyama and colleagues investigated changes in endothelial function by linagliptin treatment compared to placebo and metformin treatment in subjects with type 2 diabetes and without macrovascular disease. While there was a significant improvement within the linagliptin group from baseline to study end, they were not able to demonstrate a significant improvement in the change of endothelial function compared to the other two groups [23]. The recent EFFORT trial is very similar to our trial in design, investigating subjects with early type 2 diabetes and coronary artery disease. They demonstrate an improvement in endothelial function measured by reactive hyperaemia-peripheral arterial tonometry by a 3 months linagliptin treatment compared to a voglibose group [24]. The major limitation of this small trial (in total 16 subjects) is the open label design without providing details on a prespecified sample size calculation in the publication.

The neutral effect shown by our study fits well into the framework of the neutral cardiovascular outcome trials seen with compounds of this group [4–6]. It is also in line with the neutral results of the recently published MARLINA-T2D trial, which did not confirm previous suggestions of an improvement in acute glomerular damage measured by the urinary albumin-creatinine ratio in subjects with type 2 diabetes [25]. The novelty of our study is that compared to in particular cardiovascular outcome trials, where usually subjects with longer diabetes duration are included, we performed our study solely in subjects with earlier stages of very well controlled diabetes and established coronary artery disease, given that previous analyses were suggesting that with increasing diabetes duration interventions are less likely to have an impact on endothelial function [26]. However, our data suggest, that also in this patient group linagliptin has a neutral effect on endothelial function. Future analyses using novel-omics techniques such as metabolomics might help in finding biomarker patterns to select patient subgroups still benefiting of a particular treatment despite neutral outcome in the overall population [27].

Data from a small ancillary study performed within the main trial did not show any changes in stem cell counts by linagliptin supporting a safe drug profile.

One limitation of our study is that we compared linagliptin against placebo and not an active control drug. However, given that the mean HbA1c at baseline was below 53 mmol/mol, the impact of linagliptin on glucose lowering was statistically significant, but very limited in the extent, as one would expect. Therefore, the glucose lowering effect observed in the linagliptin group is very unlikely to have an influence on endothelial function. Another limitation is the trial duration of 3 months. One could argue that a longer treatment period might be necessary to demonstrate a benefit on endothelial function in a cohort already having coronary atherosclerosis. However, our neutral results fit well in the overall neutral cardiovascular effects observed for DPP-4 inhibitors in recent cardiovascular outcome trials [4–6]. Further, the overall number of subjects included, being 43, could be considered small, however, the trial was powered to demonstrate a potential relative 30% improvement in FMD, which is deemed a clinically meaningful improvement.

## Conclusion

Our study in subjects with early diabetes and established coronary artery disease demonstrates a neutral effect of linagliptin on various measurements of endothelial function. Whether this finding is also true for subjects with newly diagnosed diabetes without established cardiovascular disease, needs further investigation.

## Additional file

**Additional file 1: Table S1.** Change in cell counts from baseline in linagliptin vs. Placebo groups. FMD: flow mediated dilatation; MTT: meal tolerance test; sVCAM-1: soluble vascular cell adhesion molecule-1; sICAM-1: soluble intercellular adhesion molecule-1.

## Abbreviations

AOR: arginine-to-ornithine-ratio
AUC: area under curve
CAD: coronary artery disease
DPP-4: dipeptidylpeptidase-4
EPC: endothelial progenitor cells
FMD: flow mediated dilatation
GABR: global arginine bioavailability ratio
GLP-1: glucagon like-peptide-1
HbA1c: glycated haemoglobin
ITT: intention-to-treat
LG: linagliptin
MSC: mesenchymal stromal cells
MTT: meal tolerance test
PG: placebo group
sVCAM-1: soluble vascular cell adhesion molecule-1
sICAM-1: soluble intercellular adhesion molecule-1
T2DM: type 2 diabetes mellitus
