# Supplementary material for: Effects of linagliptin on endothelial function and postprandial lipids in coronary artery disease patients with early diabetes: a randomized, placebo-controlled, double-blind trial
Source: Cardiovasc Diabetol. 2018 May 17;17:71. doi: 10.1186/s12933-018-0716-x (PMC5958406; doi:10.1186/s12933-018-0716-x)
Supplement: Supplementary file 1 — Additional file 1: Table S1. Change in cell counts from baseline in linagliptin vs. Placebo groups. FMD: flow mediated dilatation; MTT: meal tolerance test; sVCAM-1: soluble vascular cell adhesion molecule-1; sICAM-1: soluble intercellular adhesion molecule-1. [file 12933_2018_716_MOESM1_ESM.docx]

Additional file 1: Table S1: Change in cell counts from baseline in Linagliptin versus Placebo groups

|  | Linagliptin  Change (Baseline – Follow-up) | Placebo  Change (Baseline – Follow-up) | *P* Value |
| --- | --- | --- | --- |
|  | Median (Min - Max) | Median (Min - Max) |  |
| LMPP of CD34+ | 0.60 (-0.69 - 1.90) | -0.10 (-0.5 - 1.4) | 0.881 |
| LMPP of CD45+ | 0.40 (-7.9 - 81.5) | 55.0 (13.2 - 181) | 0.456 |
| MEP of CD34+ | -2.4 (-6.6 - -1.1) | -1.5 (-4.5 - 1.6) | 0.453 |
| MEP of CD45+ | -62.6 (-322.8 - 81.4) | 4.2 (-44.4 - 81.7) | 0.297 |
| MLP/CLP of CD34+ | 0.4 (-1.9 - 2.1) | 0.4 (0.3 - 0.7) | 0.653 |
| MLP/CLP of CD45+ | 3.6 (-15.3 - 52.3) | 34.1 (7.5 - 38.1) | 0.881 |
| MPP of CD34+ | 3.1 (0.9 - 9.1) | 3.5 (-2.2 - 4.6) | 0.297 |
| MPP of CD45+ | 88.4 (15.4 - 434.7) | 35.3 (14.9 - 212.6) | 0.180 |
| WBCs - per µl | 1500 (-3100 - 2850) | -700 (-800 - 400) | 0.230 |
